# Correction: Scientific quality of COVID-19 and SARS CoV-2 publications in the highest impact medical journals during the early phase of the pandemic: A case control study

**DOI:** 10.1371/journal.pone.0250141

**Published:** 2021-04-08

**Authors:** Marko Zdravkovic, Joana Berger-Estilita, Bogdan Zdravkovic, David Berger

In the Results subsection of the Abstract, there is an error in the second sentence. The correct sentence is: The 155 COVID-19 publications were 18-fold more likely to be of lower evidence (95% confidence interval [CI] for odds ratio, 7.0–47; P <0.001).

In the Results subsection of the Abstract, there is also an error in the fourth sentence. The correct sentence is: There was a significant difference in the early citation rate of the original articles that favored the COVID-19 original articles (median [interquartile range], 149 [79–512] vs. 3 [1.3–7] citations; P <0.001).
