# Supplementary material for: Scientific quality of COVID-19 and SARS CoV-2 publications in the highest impact medical journals during the early phase of the pandemic: A case control study
Source: PLoS One. 2020 Nov 5;15(11):e0241826. doi: 10.1371/journal.pone.0241826 (PMC7643945; doi:10.1371/journal.pone.0241826)
Supplement: S1 File — Description of data: Detailed criteria are shown for the quality assessment of the quantitative studies. (DOCX) [file pone.0241826.s001.docx]

**S1 File**

**eTable 1.** Checklist used for the assessment of the quality of the quantitative studies^1^.

| **Item N°** | **Item description** | **Yes**  **(2 points)** | **Partial (1 point)** | **No**  **(0 points)** | **Not applicable** |
| --- | --- | --- | --- | --- | --- |
| 1 | Question/ objective sufficiently described? |  |  |  | Always  applicable |
| 2 | Study design evident and appropriate? |  |  |  | Always  applicable |
| 3 | Method of subject/ comparison group selection *or* source of information/ input variables described and appropriate? |  |  |  |  |
| 4 | Subject (and comparison group, if applicable) characteristics sufficiently described? |  |  |  | Always  applicable |
| 5 | If interventional and random allocation was possible, was it described? |  |  |  |  |
| 6 | If interventional and blinding of investigators was possible, was it reported? |  |  |  |  |
| 7 | If interventional and blinding of subjects was possible, was it reported? |  |  |  |  |
| 8 | Outcome and (if applicable) exposure measure(s) well defined and robust to measurement/ misclassification bias? Means of assessment reported? |  |  |  |  |
| 9 | Sample size appropriate? |  |  |  |  |
| 10 | Analytic methods described/ justified and appropriate? |  |  |  |  |
| 11 | Some estimate of variance is reported for the main results? |  |  |  |  |
| 12 | Controlled for confounding? |  |  |  |  |
| 13 | Results reported in sufficient detail? |  |  |  | Always  applicable |
| 14 | Conclusions supported by the results? |  |  |  | Always  applicable |
