# Supplementary material for: Scientific quality of COVID-19 and SARS CoV-2 publications in the highest impact medical journals during the early phase of the pandemic: A case control study
Source: PLoS One. 2020 Nov 5;15(11):e0241826. doi: 10.1371/journal.pone.0241826 (PMC7643945; doi:10.1371/journal.pone.0241826)
Supplement: S2 File — Description of data: Percentage assessor agreement after independent individual scoring and following resolution of disagreements. (DOCX) [file pone.0241826.s002.docx]

**S2 File.**

**eTable 2**. Assessor (authors MZ–DB, JBE–BZ) agreements on the qualities of the quantitative studies after independent individual scoring and the following resolution of disagreements.

| **Item N°** | **Item description** | **After scoring (%agreement)** | | **After resolution (%agreement)** | |
| --- | --- | --- | --- | --- | --- |
|  |  | **MZ–DB** | **JBE–BZ** | **MZ–DB** | **JBE–BZ** |
| 1 | Question/ objective sufficiently described? | 87.5 | **60.6** | **90.6** | **72.7*** |
| 2 | Study design evident and appropriate? | 90.6 | **72.7** | **90.6** | **81.8*** |
| 3 | Method of subject/ comparison group selection *or* source of information/ input variables described and appropriate? | 90.6 | **66.7** | **96.9** | **81.8** |
| 4 | Subject (and comparison group, if applicable) characteristics sufficiently described? | 90.6 | **69.7** | **90.6*** | **78.7*** |
| 5 | If interventional and random allocation was possible, was it described? | **78.1** | 97 | 81.3 | 97.0 |
| 6 | If interventional and blinding of investigators was possible, was it reported? | **65.7** | 84.9 | 78.1 | 87.9 |
| 7 | If interventional and blinding of subjects was possible, was it reported? | **68.8** | 80.9 | 81.3 | 93.9 |
| 8 | Outcome and (if applicable) exposure measure(s) well defined and robust to measurement / misclassification bias? Means of assessment reported? | 84.4 | **69.7** | **90.6** | **78.8** |
| 9 | Sample size appropriate? | **59.3** | **78.8** | **75.0*** | **84.8** |
| 10 | Analytic methods described/ justified and appropriate? | 87.5 | **75.7** | **96.9** | **87.9** |
| 11 | Some estimate of variance is reported for the main results? | 90.6 | 84.8 | 100.0 | 90.9 |
| 12 | Controlled for confounding? | **43.7** | **30.3** | **56.2*** | **51.5*** |
| 13 | Results reported in sufficient detail? | 84.4 | 90.9 | 87.5* | 90.9 |
| 14 | Conclusions supported by the results? | 87.5 | **78.8** | **87.5*** | **78.8*** |
|  | ***Kappa <0.5 (N)** | N/A | N/A | 5 | 4 |
|  | **Total score (Cronbach’s alpha)** | 0.930 | 0.962 | 0.987 | 0.988 |
|  | **Summary percentage score (Cronbach’s alpha)** | 0.866 | **0.696** | **0.964** | **0.928** |

N/A, not applicable
