# Supplementary material for: Scientific quality of COVID-19 and SARS CoV-2 publications in the highest impact medical journals during the early phase of the pandemic: A case control study
Source: PLoS One. 2020 Nov 5;15(11):e0241826. doi: 10.1371/journal.pone.0241826 (PMC7643945; doi:10.1371/journal.pone.0241826)
Supplement: Attachment — Submitted filename: Reply to Reviewers for PONE-R2.docx [file pone.0241826.s004.docx]

Reply to Reviewers for PONE-D-20-14688R1:

Scientific Quality of COVID-19 and SARS CoV-2 Publications in the Highest Impact Medical Journals during the Early Phase of the Pandemic: A Case Control Study

Review Comments to the Author

Reviewer #1: Many thanks for the opportunity to review the revised article. This remains good research, fit for publication, and I believe the authors have mostly addressed the issues discussed during the prior round of review. I offer 1 major issue and some additional brief comments and replies to the author’s responses. Assuming these are addressed, I would recommend for publication.

***Our reply:*** *We thank the reviewer for the appreciation of our work*

Major Issues:

My biggest remaining issue is the “Qualitative evaluation.” I thank the authors for their explanation of mixed-methods research but to claim you used a “mixed-methods methodology” that includes a “Qualitative” section you need to actually include and describe some sort of qualitative method used to evaluate the work. e.g., thematic analysis, grounded theory, content analysis, framework analysis etc. These are all well established, detailed, systematic methods for conducting qualitative analysis. A one sentence qualitative methods section that just states that the research was “assessed qualitatively” is not sufficient. The Cochrane Risk of Bias tool may have provided a deductive framework around which to categorise and report these evaluations (using something like content analysis) is what I was getting at. You should remove the word “Qualitative” from this section and replace it with something like “Narrative” pr “Subjective” assessments so as to not give the impression that a robust, systematic Qualitative method was used for this evaluation. At least, based on what is reported in the paper, that does not seem to be the case.

***Our reply:*** *Thank you for this comment. We have changed the wording “qualitative” to “narrative” throughout.*

Minor Issues:

Many thanks to the authors for addressing how they selected the journals. They may be interested to see, if they have not already, that some very similar research was presented recently at the European Society of Cardiology Congress 2020. <https://www.tctmd.com/news/covid-19-blamed-weaker-research-published-top-tier-journals-2020> The fact that they looked at the same three journals, however using different criteria/scales and historic controls. This is an interesting check on the findings from Zdravkovic et al. and can be brought out in the discussion. I couldn’t locate a book of abstracts for that conference (it may not be available yet) but the results are described in that article. Obviously not ideal for referencing, but the similarity is notable and probably worth being mentioned in relation to this research.

***Our reply:*** *This is indeed very interesting. Thank you for making us aware. We have contacted Professor Stefanini and he provided us the slideset that he presented at the congress. He states that besides that, his data do not exist in a published and citable form yet. We have included a statement in the discussion. If this does not suit your citation policy, please advise us.*

“One might argue that a comparison to a historical control group, for example the same time frame in 2019, when there was no pandemic effect on research, would have been more appropriate. Our hypothesis was that COVID-related research showed lower quality than non-COVID research. A historical control group may introduce a selection bias, since conditions for research then would be clearly different. We would therefore argue that the control group has to be subject to the same conditions as the test group, when methodological quality is assessed. This may be different for other endpoints like total research output. In line with our results, Stefanini et al reported - in an oral presentation at the European Society of Cardiology Congress 2020 - similar findings of lower quality associated with COVID-19 in the same journals and time frame as our work with a historical control group of 2019. So, both historical and contemporary control groups lead to the same conclusions.”

I am glad to see the sensitivity analysis removing editorials. I disagree that ~8% and ~14% of your sample is very small. That said it is very good that your findings remained robust to removing these. I think that your findings remained robust to the various sensitivity analyses is a strength.

***Our reply:*** *Thank you*

I don’t think it is correct to say that the JAMA Research Letter format (or other journal’s similar formats) are indicative of the editors/reviewers believing the articles “are not good enough to meet the criteria for Original article category.” You can submit directly in the Research Letter format without being referred there by the editors. Some research simply doesn’t need 2000+ words to get the point across. That said, I think the inclusion of the sensitivity analysis is sufficient.

***Our reply:*** *Thank you*

On the citation analysis, I agree that with the new data, it would be unreasonable to check all of these citations for context. However I do think the levels originally reported through May could reasonably have been investigated. I understand that you feel this is out of the scope of this paper and resources of your group and respect the decision not to investigate further at this time. I would simply request that if you agree that what I stated about citation context may be true, that it is mentioned as relevant to the interpretation of this finding on Page 14 and potentially as a direction for future research in the Discussion. A case study in this that has personally annoyed me quite a bit is Didier Raoult going on about how many times his hydroxychloroquine paper has been cited, as a defense of the paper, with no context of how many of those were citing it in the context of pointing out the many limitations of that research. No need to use this example but I think it proves the point.

***Our reply:*** *We fully agree with your notion and have expanded the discussion:*

It must be acknowledged though, that a citation does not necessarily need to be positive for a study or author, if the context, i. e. criticisim or discussions about retractions and corrections, of the citations are considered. This is beyond the scope of our work.

I would like to applaud the authors for making their data openly available.

***Our reply:*** *Thank you*

Many thanks again for the opportunity to review this paper. If these issues can be rectified to the editor’s satisfaction I am happy to recommend this paper go forward to publication.

***Our reply:*** *We thank you for your constructive criticism and hope that we have now sufficiently addressed all the points raised.*

Reviewer #2: 1. I would like to comment on the following author’s answer:
A-2-3: We fully agree with the reviewer. We had planed an observational study design as a case control. We were then obliged by PLOS ONE to include “systematic review” in the title, because we deal with study comparisons. We would like to follow your suggestion to go as a case-control study which was the initial plan, unless the editorial office overrules us.
Actually, I agree that this is not a systematic review therefore I would avoid any reference to this study design in order to not arise misunderstanding regarding the study type. I did not find the term “systematic review in the title accordingly and I agree with the removal of any reference to the PRISMA statement.

***Our reply:*** *Thank you for supporting our idea. We have already changed the title according to your suggestion and journal editors seem to agree.*

2. My major concern is still related to the quality appraisal the authors performed. I went through the references the authors reported to support the choice of QUALSYST tool use, the mixed methodology and still some issues and confusion raised.
I agree with their comment A1-6 B where the authors described the definition of qualitative and quantitative research but I think something is not still clear or there is some confusion in the description of this concept. They stated: Qualitative analysis is the analysis of qualitative data such as text data from interview transcripts. Unlike quantitative analysis, which is statistics-driven and largely independent of the researcher, qualitative analysis is heavily dependent on the researcher’s analytic and integrative skills and personal knowledge of the social context where the data is collected. The emphasis in qualitative analysis is “sense making” or understanding a phenomenon, rather than predicting or explaining. This methodology is very frequently used in social sciences in combination with quantitative analysis, the so called mixed-methods methodology.
I am aware regarding the mixed-method methodology but I don’t think this is the case to adopt it: here the author should have assessed just the methodological quality of the quantitative research study design (ie. experimental, observational etc….) offering a qualitative assessment and not a quantitative one which in the Cochrane wording is referring to a quantitative synthesis/statistically driven. Moreover, the second part the authors assessed, ie. “qualitative analysis” is just an assessment of reporting characteristic of the included studies - I did not assess it as a qualitative analysis. PLease look at the following examples of reporting characteristics assessment/methdoological quality: <https://journals.plos.org/plosmedicine/article?id=10.1371/journal.pmed.0040078>; <https://www.sciencedirect.com/science/article/pii/S0895435616308162>.
Otherwise the study design is not clearly described – the mixed methodology might describe the study design and not the appraisal of the studies. Here an example: <https://pubmed.ncbi.nlm.nih.gov/33033951/> where both a questionnaire(quantitative analysis) and focused groups (qualitative analysis) were performed.

In this case control study, this mixed methodology did not really reflect what was done. To my opinion is a control study belonging to the quantitative research world where included studies were compared using the following instruments:
1. Methodological quality throughout the QUALSYST tool (even though the high standard: Cochrane Risk of bias or the New Castle or the ROBINS I could have been used too)
2. Asssessment of reporting elements such as: reported from page 7, lines 150: ”The COVID-19 original research articles (n = 13) were assessed qualitatively to report on their major weaknesses (which type of weakness and how this is standardized across studies? Was not already included in the methodological quality for quantitative studies in the QUALSYST tool?), potential conflicts of interest, and likely influence on further research and clinical practice (in which way these are standardized, collected and reported in the assessment? Are regression analyses planned to investigate the influence? ).
The second point cannot be equal to a qualitative part of a mixed methodology where usually focus groups or interviews are used to collect qualitative data. The data/info the authors wanted to comment on are included and reported in the selected studies, in the manuscript/full text as general characteristics (ie. conflict of interest) and so are simply collected from them and then discussed.
I suggest the authors to better revise the study design performed, the qualitative/quantitative wording.

***Our reply:*** *Thank you. As suggested by reviewer 1, we changed the wording “qualitative” to “narrative” throughout the manuscript. We also changed the “Mixed-methods” wording to “multi-step”, to more accurately reflect the methodology used.*
